# Preclinical pharmacokinetics, pharmacodynamics, and efficacy of RG7116: a novel humanized, glycoengineered anti-HER3 antibody

**DOI:** 10.1007/s00280-015-2697-8

**Published:** 2015-02-22

**Authors:** Georgina Meneses-Lorente, Thomas Friess, Irene Kolm, Gabriele Hölzlwimmer, Sabine Bader, Christophe Meille, Marlene Thomas, Birgit Bossenmaier

**Affiliations:** 1 Pharma Research and Early Development, Clinical Pharmacology, Roche Innovation Center Welwyn, Welwyn Garden City, UK; 2 Pharma Research and Early Development, Roche Innovation Center Penzberg, Penzberg, Germany; 3 Translational Technologies and Bioinformatics, Roche Innovation Center Penzberg, Penzberg, Germany; 4 Department of PL and Modeling and Simulation, Pharmaceutical Sciences (PS), Roche Innovation Center Basel, Basel, Switzerland

**Keywords:** RG7116, GE-huMab-HER3, HER3, Signaling inhibition, Pharmacokinetics, Pharmacodynamics

## Abstract

**Purpose:**

RG7116 is a novel anti-HER3 therapeutic antibody that inhibits HER3 signalling and induces antibody-dependent cellular cytotoxicity of tumor cells due to a glycoengineered antibody Fc moiety. We investigated the efficacy and pharmacokinetic/pharmacodynamic properties of HER3 signal inhibition by RG7116 in a murine xenograft model of human head and neck cancer.

**Methods:**

SCID-beige mice bearing FaDu cells were treated with RG7116 at a weekly dose of 0.3–10 mg/kg, and tumor growth control and modulation of selected proteins (HER3 and AKT) were examined.

**Results:**

Complete tumor stasis up to Day 46 was observed at a dose >3 mg/kg, and this dose down-modulated membrane HER3 expression and inhibited HER3 and AKT phosphorylation. Systemic RG7116 exposure was greater than dose-proportional and total clearance declined with increasing dose, indicating that RG7116 elimination is target-mediated. This is consistent with the better efficacy, and the HER3 and pAKT inhibition, that was observed at doses >1 mg/kg. Tumor regrowth occurred from Day 46 onwards and was associated with HER1 and HER2 upregulation, indicating the activation of alternative HER escape pathways. Modulation of HER3 and phospho-HER3 was also demonstrated in the skin and mucosa of an RG7116-treated cynomolgus monkey, suggesting that these may be useful surrogate tissues for monitoring RG7116 activity.

**Conclusions:**

These data confirm the promising efficacy of RG7116 and highlight the value of assessing the PK behavior of the antibody and measuring target protein modulation as a marker of biological activity. Clinical development of RG7116 has now begun, and phase I trials are ongoing.

## Introduction

The human epidermal growth factor receptor 3 (HER3/ERBB3) is a 185-kDa member of the evolutionary-conserved family of HER transmembrane receptors. Together, these four receptor tyrosine kinases form a dynamic signaling network that transduces extracellular growth signals into the cell and activates multiple cellular pathways involved in proliferation, cell survival, and differentiation. HER receptors normally exist as inactive monomers [1] and only become activated in response to overexpression or ligand binding followed by receptor dimerization. HER3 contains an extracellular neuregulin-binding domain, but unlike the other members of the HER family, it lacks an active intracellular kinase domain. Therefore, HER3 signaling is mediated through heterodimerization with other HER members. The HER2(ERBB2)/HER3 heterodimer is among the most stable of HER dimers and is a particularly potent initiator of phosphoinositide 3-kinase (PI3K) signaling [1]. Once activated, PI3K then triggers the translocation of AKT to the phospholipid membrane—through the second messenger PIP3—where AKT becomes phosphorylated and acts as a kinase for multiple substrates [2].

The role of HER1 (EGFR) and HER2 in tumorigenesis is well characterized, and multiple therapeutic compounds targeting these members of the HER family have now been developed. In contrast, less attention has been paid to the role of HER3 in human cancer. Elevated expression of HER3 is seen in many solid tumor types, and upregulation of HER3 is associated with poor outcome and reduced survival [3–8]. Indeed, HER3 may be required for the oncogenic transformation of normal cells by other HER members [9, 10]. Activated HER3 can interact directly with the p85 subunit of PI3K, whereas HER2 and HER1 cannot [11]. Furthermore, upregulation of HER3 in response to therapeutic inhibition of other HER members is a recognized mechanism by which tumor cells can escape the action of HER1- and HER2-targeted therapies [12, 13]. Consequently, several antibodies targeting HER3 are now under investigation [14–21].

RG7116 is a novel humanized anti-HER3 therapeutic monoclonal antibody (mAb) with a dual mode of action. RG7116 binds selectively to domain 1 of HER3 with high affinity and effectively prevents binding of the HER3 ligand, heregulin [22]. Binding of RG7116 to the extracellular domain (ECD) of HER3 is characterized as a fully enthalpy-dominated key-and-lock mechanism, typical for a fully mature antibody–antigen interaction [23, 24]. HER3 bound to RG7116 is maintained in the inactive, unliganded conformation, and the high stability of this complex effectively prevents subsequent phosphorylation of HER3 and AKT phosphorylation in vitro. This translated into a potent inhibition of tumor cell growth in vitro, and robust efficacy in a variety of murine subcutaneous xenograft models of human cancer [22]. In addition to the therapeutic efficacy derived from HER3 signaling inhibition, RG7116 is glycoengineered for enhanced antibody-dependent cellular cytotoxicity (ADCC) [22].

In this study, we investigated the efficacy and pharmacokinetics (PK) of RG7116 treatment using a subcutaneous mouse xenograft model of human hypopharyngeal cancer. In addition, the expression of pharmacodynamic (PD) markers of HER3 signaling in response to RG7116 therapy was also investigated.

## Materials and methods

### Cell lines

FaDu human hypopharyngeal squamous cell carcinoma cells (HER1 and *KRAS* wild type) were obtained from the American Type Culture Collection. Cell lines obtained from these suppliers are routinely authenticated by karyotyping, short-tandem repeat profiling, assessment of cell morphology, and species verification by isoenzymology. Cell lines were expanded upon receipt and aliquots frozen. Cells were not passaged for more than 6 months after resuscitation. Tumor cells were routinely cultured in MEM medium supplemented with 10 % fetal bovine serum, 2 mM l-glutamine, 1× NEAA, and 1 mM sodium pyruvate at 37 °C in a water-saturated atmosphere and 5 % CO_2_. Culture passage was performed with 0.05 % trypsin and 0.02 % EDTA in phosphate-buffered saline every sixth–seventh day. All reagents were obtained from PAN Biotech GmbH, Germany.

### Xenograft model

FaDu cells (5.0 × 10^6^ cells/mL) were injected subcutaneously under anesthesia into the right flank of female SCID-beige mice (CB17.Cg-PrKdc^scid^Lyst^bg^; age 5–6 weeks at arrival; Charles River, Germany). After inoculation, FaDu xenograft tumors displayed rapid progressive growth (take rate 100 %) with an in vivo tumor doubling time of 2–3 days. Mice were maintained under specific-pathogen-free condition with daily cycles of 12-h light/12-h darkness according to the guidelines (GV-Solas; Felasa; TierschG) with food, and water was provided ad libitum. All animal experiments were conducted according to the guidelines of the German Animal Welfare Act and were approved by local government. Animals were examined daily for clinical symptoms, detection of adverse effects, and assessment of body weight. Mice were randomized on Days 14–18 when tumor volume was approximately 200 mm^3^ and treatment started immediately.

Study FaDu_001: FaDu-bearing SCID-beige mice (*n* = 9–10/group) were randomized on Day 14 after inoculation (mean tumor volume [TV] 150 mm^3^), and RG7116 was administered at dose levels of 0.3, 1.0, 3.0, and 10 mg/kg weekly ip on Days 14, 21, and 28. Serum was collected before the second (Day 21) and last treatment (Day 35) together with tumor (Day 35). Study FaDu_006: Mice were randomized on Day 17 (mean TV 220 mm^3^), and RG7116 was administered as a single i.v. bolus dose at 0.3 and 1 mg/kg on Day 18 (*n* = 35 mice/dose group; *n* = 15/control group). Serum and tumor were collected 1, 3, 6, 24, 96, 168, and 240 h after RG7116 injection from five mice/time point/group (controls: 1, 96, 168 h). Study FaDu_008: 15 mice/group (mean TV 180 mm^3^) were randomized on Day 18, and RG7116 was given weekly i.v. at 0.3 and 3.0 mg/kg on Days 18, 25, 32, 39, 46, 53, and 60 (or 3.0 mg/kg q3w on Days 18 and 39). Serum and tumor samples were collected on Days 25, 32, and 39 (five mice/time point). The 0.3 mg/kg dosing group was rechallenged with RG7116 at 5 mg/kg on Days 39, 46, 53, and 60.

### Assessment of anti-tumor efficacy

Tumor volume (TV) was measured by caliper twice-weekly beginning at randomization according to a standard formula (TV = ½ length × width^2^); values were documented as means and standard error of the mean (SEM). Tumor growth inhibition (TGI) during the treatment period was calculated by comparing each treated group (treated) with its respective vehicle-treated control (resp. control) using the formula:

$${\text{TGI}}\; ( {\text{\%)}} = 100 - \frac{{\overline{\text{TV}} \left( {\text{treated}} \right)_{{{\text{day}}\;x}} - \overline{\text{TV}} \left( {\text{treated}} \right)_{{{\text{day}}\;y}} }}{{\overline{\text{TV}} \left( {{\text{resp}}.\;{\text{control}}} \right)_{{{\text{day}}\;x}} - \overline{\text{TV}} \left( {{\text{resp}}.\;{\text{control}}} \right)_{{{\text{day}}\;y}} }} \times 100$$

where $\overline{\text{TV}}_{{{\text{day}}\,x}}$ represents the average tumor volume of a study group on study day *x*.

Data regarding tumor growth were analyzed using nonparametric methods, since the data showed asymmetrical behavior. Prior to this procedure, the data were baseline-corrected with the tumor volume at the of the start treatment.

### Pharmacodynamic analysis of the effect of RG7116 on target modulation

#### Immunohistochemistry

Tissue was formalin-fixed and paraffin-embedded, and standard immunohistochemistry (IHC) was conducted using a murine mAb specific for HER3 (clone DAK-H3-IC, DAKO, #M7297) and rabbit mAb specific for phosphorylated HER3 (pHER3; clone 21D3, Cell Signaling Technologies, #4791). Staining was conducted using the DAKO autostainer (HER3) or Ventana Benchmark XT system (pHER3).

#### Western Blot analyses

Tissue was immediately lysed using Triton Lysis Buffer (1 % Triton-X-100; 10 µg/mL aprotinin; 0.4 mM orthovanadate; 1 mM phenylmethylsulfonyl fluoride), and lysates were denatured in NuPAGE Sample Reducing Agent at 70 °C for 10 min. SDS–PAGE and Western blotting were conducted as described previously [22] using 20 μg protein per lane measured using a bicinchoninic acid assay and antibodies specific for HER1 (Upstate/Millipore, #06-847), HER2 (Dako, #A485), HER3 (clone C-17, Santa Cruz, #sc-285), phosphorylated HER1 (pHER1, clone ID Y39; Epitomics/Abcam, #ab32086), or pHER3 (clone 21D3 [Tyr^1289^], Cell Signaling Technologies, #4791).

#### ELISA

Inhibition of AKT phosphorylation was examined in FaDu tumor lysates using the anti-phospho-AKT (pSer^473^) enzyme immune assay kit according to the manufacturer’s instructions (Enzo Life Sciences). Briefly, cell lysates and reference standards were incubated in wells coated with antibody specific for the amino terminus of AKT. Plates were washed and further incubated in biotinylated antibody specific for AKT phosphorylated at Ser^473^. After another wash, signal was detected using a solution of streptavidin-HRP conjugate and tetramethylbenzidine substrate and quantified in a spectrometer at 450 nm. The amount of signal was directly proportional to the level of AKT 1/2 pSer^473^ in the sample.

#### Gene expression profiling

A portion of tumor was preserved in RNAlater (Qiagen) for gene expression analysis. 50 ng of total RNA was amplified using the NuGen Ovation kit followed by labeling with the NuGen Encore kit. Gene expression profiling for HER1 (232541_at) and ERBB2 (probe set 216836_s_at) was performed on U133 plus 2.0 Genechips (Affymetrix). Preprocessing of the mRNA data was performed using the statistics software R (version 2.13.2), running in house scripts for quality control and data consolidation via the robust multi-array average method.

#### Analysis of oral mucosa and skin in cynomolgus monkeys

Pharmacodynamic analyses in cynomolgus monkeys were conducted at Covance under standard operating procedures and in compliance with applicable regulations about the use of laboratory animals. Skin and buccal mucous membrane biopsies were collected once before dosing i.v. 20 mg/kg RG7116 and at approximately 2 and 6 h after dosing. A skin patch of approximately 1 g (approximately 40 × 10 mm) was excised from the back, and a mucosal patch of approximately 0.5 g (approximately 25 × 7 mm) was excised from the buccal mucosa. Biopsies were performed under ketamine and xylazine and cut into four pieces for further preparation. The wound was closed with surgical suture, and flunixin was given as antiphlogistic after biopsy. Samples were lysed, and HER3 levels were assessed by Western Blot or pHER3 IHC as described.

### Pharmacokinetic analysis of serum RG7116 levels

The concentration of RG7116 in mouse serum was measured using a generic human IgG ELISA (lower limit of quantification: 8 ng/mL) [25]. Serum concentration–time profiles were used to estimate the following PK parameters in mouse using non-compartmental analysis (WinNonlin, version 6.2; Pharsight Corporation, Mountain View, CA, USA): total drug exposure defined as area under the serum concentration–time curve extrapolated to infinity (AUC_inf_), total clearance (CL_total_), and observed maximum serum concentration (*C*_max_). A naïve pooled approach was used in mouse to provide one estimate for each dose group.

## Results

### RG7116 inhibits tumor growth in a dose-dependent manner in FaDu-bearing SCID-beige mice

**Fig. 1:**
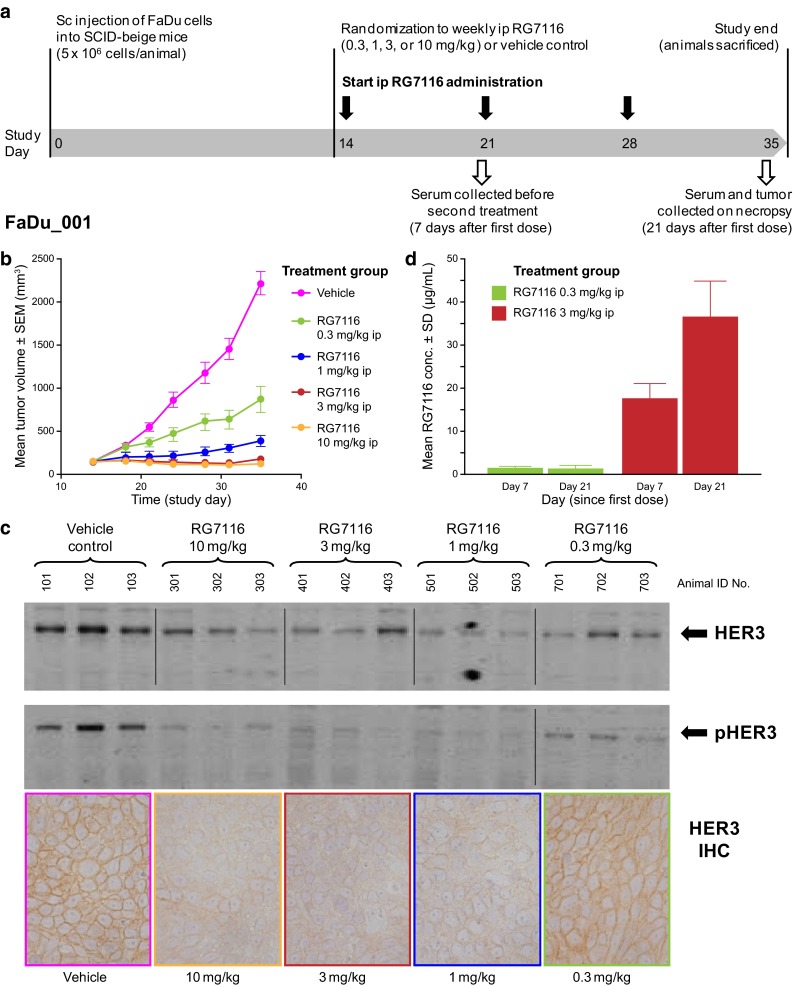
**a** Dose–response study (FaDu_001) investigating the efficacy, pharmacodynamics, and pharmacokinetics of three-weekly ip doses of RG7116 (0.3–10 mg/kg) in female SCID-beige mice bearing subcutaneous human hypopharyngeal cancer FaDu cells. **b** Dose-dependent tumor growth inhibition was observed in mice treated with three doses of weekly RG7116. **c** Expression of HER3 and pHER3 was down-modulated in mice receiving RG7116 at doses above 1 mg/kg. **d** Accumulation of RG7116 was seen with doses above 1 mg/kg but not with the 0.3 mg/kg dose. *IHC* immunohistochemistry, *SEM* standard error of the mean, *SD* standard deviation

The in vivo efficacy of single-agent RG7116 was evaluated in a SCID-beige xenograft model bearing subcutaneous FaDu human head and neck cancer cells. This model was chosen to specifically investigate the inhibitory effect of RG7116 on the HER3 signaling pathway, as SCID-beige mice are severely immune-deficient; thus, the ADCC mode of action of RG7116 plays little or no role in these experiments. FaDu xenografts express moderate levels of HER3 but have a high pHER3/HER3 ratio. This was previously shown to correlate with efficacy with RG7116 [22]. Immunohistochemical analysis identified high expression levels of membrane HER1 and medium expression of HER2 at baseline. To explore the effect of different RG7116 doses on tumor growth inhibition, RG7116 was administered to FaDu-bearing mice over a dose range of 0.3–10 mg/kg (FaDu_001). Mice received three-weekly ip doses of RG7116 starting on Day 14 (Fig. 1a).

Growth inhibition was dose-dependent and reached a plateau between 3 and 10 mg/kg. Tumor stasis was sustained for the duration of the study in animals treated with 3 and 10 mg/kg RG7116 (Fig. 1b). Tumor growth was observed after the first administration in mice treated with 0.3 mg/kg and after the third administration in mice treated with 1 mg/kg, albeit at a reduced pace compared to vehicle control mice.

Immunohistochemistry and Western blotting for HER3 conducted in xenograft explants obtained at the end of the treatment (Day 35) showed that membrane HER3 expression in tumors from mice treated with 1–10 mg/kg of RG7116 appeared to be downregulated compared to animals receiving 0.3 mg/kg RG7116 or vehicle control (Fig. 1c). All tested doses of RG7116 inhibited the phosphorylation of HER3 compared to controls, as seen by Western blotting (Fig. 1c): in mice treated with 0.3 mg/kg RG7116, the level of pHER3 was reduced compared to control animals, whereas pHER3 was undetectable in explants from mice treated at doses above 1.0 mg/kg.

Assessment of RG7116 trough concentrations (*C*_min_) showed that mice administered a weekly dose of 0.3 mg/kg did not exhibit serum RG7116 accumulation from the first to third administration (Fig. 1d); mean *C*_min_ values were 1.66 and 1.48 µg/mL, respectively (Table 1a). A clear increase in mean *C*_min_ from the first to third administration was seen in mice receiving weekly doses of 1, 3, and 10 mg/kg RG7116 (Table 1a; Fig. 1d), indicating that target saturation as a surrogate of target-mediated drug disposition was being achieved from 1 to 10 mg/kg.

**Table 1:** RG7116 pharmacokinetic parameters in FaDu-bearing SCID-beige mice by non-compartmental analysis treated with (A) weekly administration of RG7116 and (B) a single dose of RG7116

Study	Dose (mg/kg) [no. of mice]	*C*_min_ (µg/mL)		
		Day 7^a^	Day 14^a^	Day 21^a^
(*A*) *Weekly administration PK*				
FaDu_001	0.3 [*n* = 9]	1.66 (15.0)	NM	1.48 (53.4)
	1 [*n* = 10]	5.12 (23.8)	NM	10.4 (21.2)
	3 [*n* = 10]	17.8 (18.9)	NM	36.7 (21.2)
	10 [*n* = 10]	58.3 (13.0)	NM	102 (24.1)
FaDu_008	0.3 [*n* = 15]	0.79 (25.3)	0.86 (55.8)	1.26 (52.0)
	3 [*n* = 15]	8.88 (15.6)	13.5 (16.9)	17.6 (23.7)

Study	Dose (mg/kg) [no. of mice]	*C*_max_ (µg/mL)	AUC_inf_ (h^a^µg/mL)	CL_total_ (mL/h)	*t*_1/2_ (h)
(*B*) *Single-dose PK*					
FaDu_006	0.3 [*n* = 35]	2.55	192	0.0312	62.4
	1 [*n* = 35]	9.73	863	0.0232	94.2
FaDu_008	3 [*n* = 15]	17.4	4811	0.0125	167

Data are mean (CV %)

AUC, area under the curve; CL_total_, total clearance; *C*_max_, peak plasma RG7116 concentration; CV %, coefficient of variation; NM, not measured; PK, pharmacokinetics; *t*_1/2_, elimination half life

^a^Day since randomization/first dose. For all PK analyses, *n* = 5 mice were analyzed per time point

### Pharmacokinetics and pharmacodynamics of RG7116 following a single dose

**Fig. 2:**
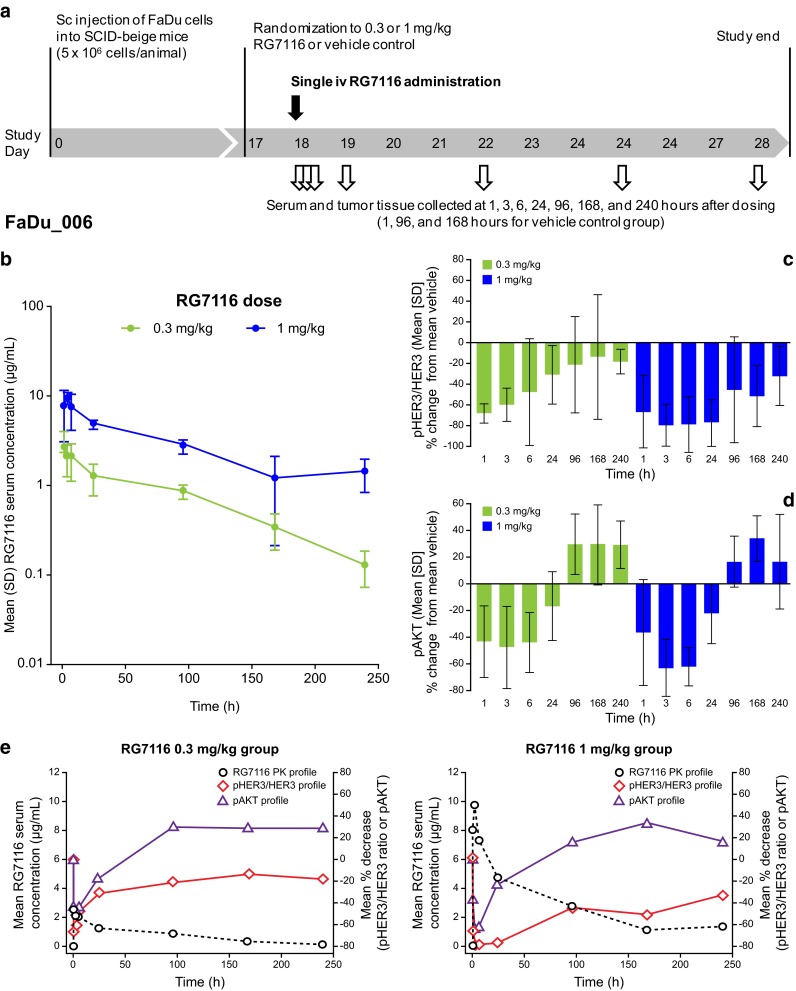
**a** Pharmacokinetics and pharmacodynamics of RG7116 following a single i.v. administration of an efficacious (1 mg/kg) and less-efficacious (0.3 mg/kg) dose of RG7116 in FaDu xenograft mice (FaDu_006). **b** PK profiles for the two doses were similar until approximately 87 h post-dose, after which rapid elimination of antibody was seen with the lower dose. **c** Inhibition of pHER3 and pAKT (**d**) was seen with both doses initially; however, this was more prolonged with the efficacious dose. **e** An inverse correlation was seen between RG7116 exposure and both pHER3 and pAKT inhibition, with faster elimination in the 0.3 mg/kg group consistent with an earlier return to baseline pHER3/HER3 levels. *SD* standard deviation

To explore the PK and PD profiles following single-dose administration and to further understand the pharmacokinetic behavior with increasing dose, a second study (FaDU_006) was conducted in which RG7116 was administered as a single i.v. bolus dose at a less-efficacious (0.3 mg/kg) and an optimal efficacious (1 mg/kg) dose, based on the results of the first study (Fig. 2a). Systemic exposure (*C*_max_ and AUC) increased with dose. However, the increase in exposure was higher than dose-proportional primarily for AUC_inf_ (Table 1b). The total clearance (CL_total_) decreased with increased dose, going from 0.0312 to 0.0232 mL/h, which is consistent with the faster elimination of RG7116 observed in the pharmacokinetic profile in the 0.3 mg/kg dose group (Fig. 2b).

The kinetics of pHER3 and HER3 inhibition following a single dose of RG7116 were investigated by Western blotting in tumor explants obtained from mice killed at 1, 3, 6, and 24 h and 4, 7, and 10 days post-treatment. Data were standardized by calculating the ratio of pHER3 to HER3 signal for each animal at each time point. Compared to controls, a maximum decrease in the mean pHER3/HER3 ratio of 66.4 % (at 1 h) and 79.5 % (at 3 h) was seen following treatment with 0.3 and 1 mg/kg, respectively (Fig. 2c). The pHER3/HER3 ratio returned to within baseline levels 96 h after treatment in mice treated with 0.3 mg/kg RG7116, whereas inhibition of HER3 phosphorylation was maintained for longer in mice treated with 1 mg/kg, with the pHER3/HER3 ratio normalizing 240 h after treatment. The higher dose of RG7116 also exerted a stronger inhibition on the downstream phosphorylation of AKT. Maximum reductions in pAKT of 47.8 and 63.6 % were observed 3 h after a single administration of 0.3 and 1 mg/kg of RG7116, respectively (Fig. 2d).

An apparent inverse correlation between RG7116 exposure and the pHER3/HER3 ratio was observed (Fig. 2e). After a single administration of 0.3 or 1 mg/kg RG7116, a rapid decrease in the pHER3/HER3 ratio was observed with a maximum mean decrease in ratio of 68.0 and 79.0 %, respectively, with the pHER3/HER3 ratio returning to baseline levels as serum RG7116 concentrations decreased. The pHER3/HER3 ratio returned to baseline values earlier in the 0.3 mg/kg treated group, consistent with the faster elimination seen in this group compared to mice treated with 1.0 mg/kg.

### Extended dosing study of RG7116

**Fig. 3:**
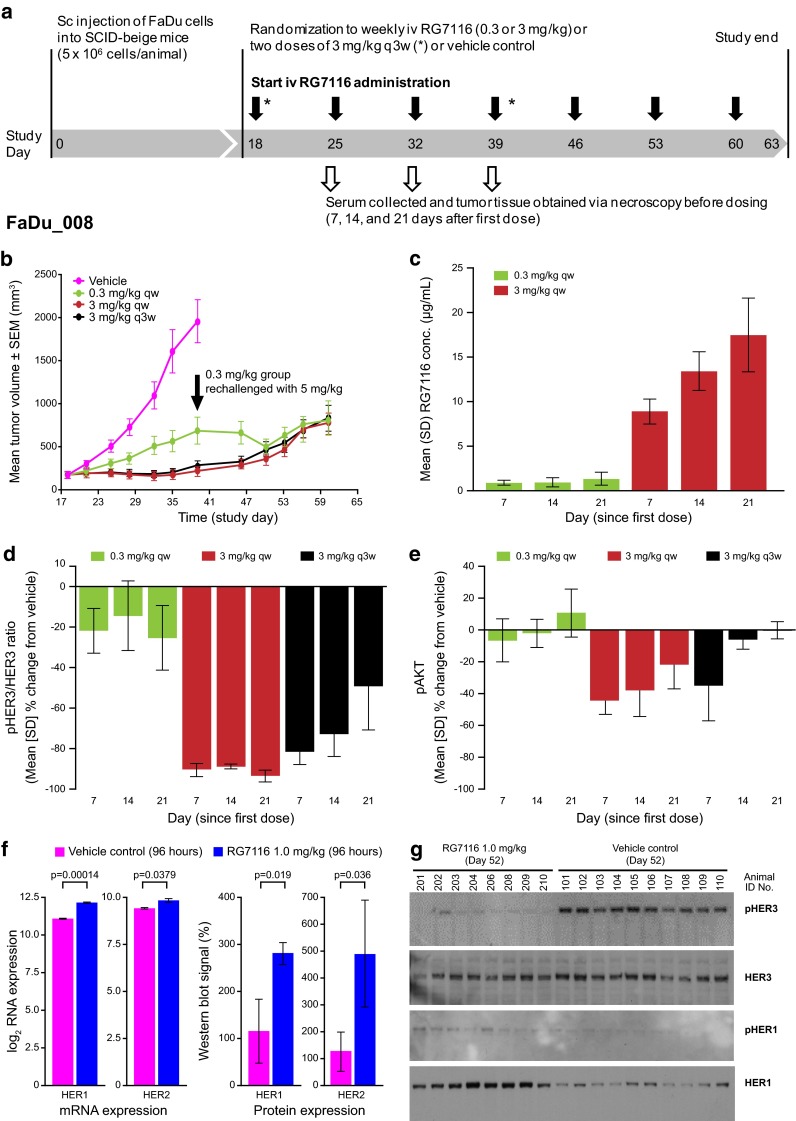
**a** Extended dosing study investigating seven i.v. weekly administrations of an efficacious dose (3 mg/kg) and a less-efficacious (0.3 mg/kg) dose of RG7116 in FaDu xenograft mice (FaDu_008). RG7116 given as a three-weekly cycle (two doses in total) was also investigated. **b** Dose-dependent efficacy up to Day 39 was seen as in the previous study and tumor growth inhibition with 3 mg/kg RG7116 given as a three-weekly cycle was similar to weekly dosing. Mice in the 0.3 mg/kg group were rechallenged (*black arrow*) with 5 mg/kg on Day 39 resulting in tumor growth inhibition for two further assessments; however, tumor regrowth was observed in all dosing groups from Day 50. **c** RG7116 trough concentrations from the first to third administrations again showed accumulation only with the higher dose. Inhibition of HER3 (**d**) and AKT (**e**) phosphorylation was also seen with weekly dosing at the efficacious dose (3 mg/kg) but not with the lower dose (0.3 mg/kg). Following a single 3 mg/kg dose (representing a three-weekly schedule), initial inhibition of HER3 phosphorylation was seen to diminish by Day 14 and 21. **f** Upregulation of HER1 (~2.1-fold) and HER2 (~1.3-fold) mRNA was seen 96 h after FaDu xenograft mice (*n* = 5) were administered 1.0 mg/kg RG7116 in a separate study, and this was associated with upregulation of HER1 and HER2 protein. **g** To compare the single animals, 20 µg total protein lysate was loaded per lane (lanes 101–110 are vehicle and 201–210 are the RG7116-treated animals). At Day 52, inhibition of HER3 phosphorylation was maintained in RG7116-treated mice, and expressed HER1 was seen to be phosphorylated compared to vehicle control. HER2 and pHER2 were not changed (data not shown). *SEM* standard error of the mean, *SD* standard deviation

To further investigate the scheduling and dose-/exposure-related anti-tumor activity of RG7116 and target modulation of HER3, an extended dosing study was performed (seven-weekly doses beginning on Day 18 after tumor implantation—study FaDu_008; Fig. 3a). A PK/PD tumor growth inhibition model (described elsewhere [26]) was used to guide the selection of the two doses investigated in this study. RG7116 was administered i.v. at an efficacious (3 mg/kg) and a less-efficacious dose (0.3 mg/kg). In addition, a group of mice were treated with a dose of 3 mg/kg once every 3 weeks (q3w) to assess the schedule dependency.

As in the first study, RG7116 showed dose-dependent inhibition of tumor growth up to Day 39 (Fig. 3b). Mice treated with 0.3 mg/kg showed tumor growth after the first administration of RG7116, whereas mice treated at the higher dose showed tumor stasis for up to 3 weeks of treatment. On Day 46, tumor regrowth was observed in mice from the 3 mg/kg groups until the end of the treatment (Fig. 3b).

Mice treated with 0.3 mg/kg of RG7116 were challenged with a higher dose of 5 mg/kg from Day 39 until the end of the treatment. This resulted in the inhibition of tumor growth for two consecutive tumor assessments (from Day 39 to Day 50) after which FaDu tumor regrowth was observed from Day 53 onwards (Fig. 3b). No clear difference in tumor inhibition and regrowth was observed between mice treated with 3 mg/kg weekly compared to 3 mg/kg q3w (Fig. 3b).

Again, no clear accumulation of RG7116 was observed with the lower dose (Table 1a; Fig. 3c). The apparent higher mean *C*_min_ value observed after the third cycle of 0.3 mg/kg RG7116 (1.26 µg/mL) was driven primarily by one mouse with higher than anticipated values. Following the administration of 3 mg/kg, a clear increase in *C*_min_ was observed (Table 1a; Fig. 3c). A full PK profile was obtained from mice treated with RG7116 3 mg/kg q3w, and a non-compartmental analysis was also conducted, which indicated that an increase in systemic exposure (AUC_inf_) was higher than dose-proportional from mice treated with 1 mg/kg (863 h*µg/mL—FaDu_006) to mice treated with 3 mg/kg (4,811 h*µg/mL—FaDu_008). This increase in AUC_inf_ was accompanied by a further decrease in total clearance from 0.0232 to 0.0125 mL/h (Table 1b). *C*_min_ values in study FaDu_008 were lower than those from mice treated with the same dose of RG7116 in study FaDu_001 (Table 1a). This may reflect the larger tumor volumes (and hence lower systemic exposure) seen in study FaDu_008 at baseline (mean 237 vs. 142 mm^3^ in study FaDu_001) and after 7 days of treatment (250 vs. 161 mm^3^ in study FaDu_001).

The pHER3/HER3 ratio and level of pAKT were analyzed in mice killed 7, 14, and 21 days after starting treatment with weekly 0.3 and 3 mg/kg RG7116 (representing animals receiving a total of one, two or three doses of RG7116, respectively). Assessment of biomarkers was also performed in animals killed 21 days after a single dose of 3 mg/kg of RG7116 (representing a q3w dosing schedule). A clear difference in the pHER3/HER3 ratio was observed between mice treated with weekly 0.3 mg/kg RG7116 and 3 mg/kg RG7116 (Fig. 3d). The pHER3/HER3 ratio was not decreased at any time point, following weekly administration of 0.3 mg/kg RG7116; however, a dramatic reduction in the pHER3/HER3 ratio was observed 7, 14, and 21 days after initiation of weekly 3 mg/kg RG7116. Clear inhibition of pHER3/HER3 was also observed after seven and 14 days with RG7116 3 mg/kg q3w; a lower pHER3/HER3 inhibition (~50 %) was observed by the end of the dosing interval (Day 21; Fig. 3d).

Similar to the pHER3/HER3 ratio, pAKT was not inhibited by the end of the weekly dosing interval when mice received the lower dose of RG7116 (0.3 mg/kg; Fig. 3e). Mice receiving weekly doses of 3 mg/kg RG7116 showed reduced levels of pAKT after the first two cycles (−44.1 and −37.9 %, respectively); however, by 21 days (after three administrations), suppression of pAKT inhibition was seen to diminish (−21.6 %; Fig. 3e). pAKT levels were initially reduced in mice receiving 3 mg/kg on a three-weekly cycle, but pAKT inhibition was diminished by 14 days after treatment initiation.

### Tumor regrowth and HER1/HER2 escape

To investigate the cause of the tumor regrowth observed after Day 46 in mice treated with an efficacious dose of RG7116 (1 mg/kg qw; Fig. 3b), mRNA and protein levels of HER1 and HER2 were examined in a separate study. Treatment with RG7116 triggered a significant upregulation of HER1 (~2.1-fold, *p* value 0.00014) and HER2 (~1.3-fold; *p* value 0.0379) mRNA and HER1 (*p* value 0.019) and HER2 (*p* value 0.036) protein levels compared to vehicle control, occurring as early as 4 days after dosing with 1.0 mg/kg RG7116 (Fig. 3f). Western blot analysis on Day 52 (Day 39 in control mice) demonstrated that the expressed HER1 was phosphorylated in RG7116-treated mice, and inhibition of HER3 phosphorylation was maintained at this time point (Fig. 3g). HER2 and pHER2 levels were not changed (data not shown).

### Exploratory pharmacokinetic and tumor growth assessment

**Fig. 4:**
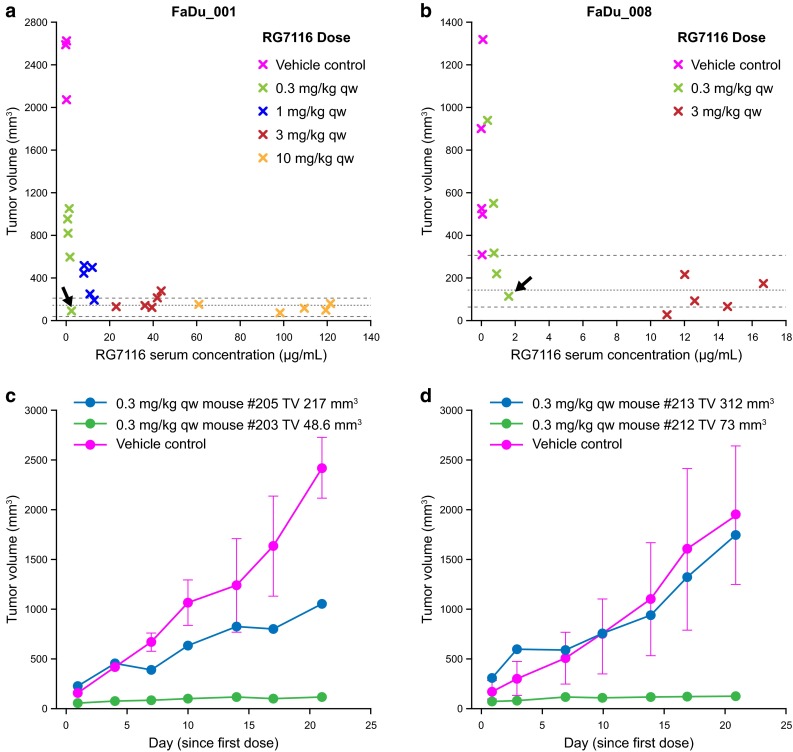
Tumor growth control following three-weekly administrations of RG7116 correlated with RG7116 exposure. In mice achieving higher serum exposure of RG7116 after 3 weeks of RG7116 treatment, tumor volume tended to reduce to baseline ranges (*dotted lines*) compared to other mice (**a** study FaDu_001 and **b** study FaDu_008). In each study, a high level of tumor growth control was observed in a mouse receiving the lowest (0.3 mg/kg) dose (*black arrows*). These mice had the smallest baseline tumor volumes in each of the studies (**c**, **d**). Comparison of the tumor growth control rate afforded by 0.3 mg/kg in the mice with the smallest and largest baseline tumor volumes in study FaDu_001 (**c**) and FaDu_008 (**d**)

In order to investigate the observed variability in response to RG7116 (seen primarily with the 0.3 mg/kg dose), an exploratory analysis of the relationship between tumor volume and RG7116 serum concentrations after three administrations was performed using five mice per group (Fig. 4a, b for study FaDu_001 and FaDu_008, respectively). A trend was observed between tumor volume and RG7116 exposure in both studies. After three administrations, tumor volumes in animals with higher RG7116 serum exposures tended to be within baseline tumor volume ranges, whereas mice achieving lower serum RG7116 exposure tended to have larger tumors. Interestingly, one mouse in each study treated with the lowest dose tested (0.3 mg/kg) also showed tumor volumes after 3 weeks of treatment within baseline ranges, comparable with mice treated with the highest doses tested in each study. These two mice had the lowest baseline tumor volumes in each of the studies (48.6 and 73.0 mm^3^ for FaDu_001 and FaDu_008, respectively). Comparison of the effect on tumor growth in mice with the largest and smallest baseline tumor volumes treated with 0.3 mg/kg is shown in Fig. 4c, d for studies FaDu_001 and FaDu_008, respectively. Notably, tumor growth in control-treated mice was not dependent on baseline tumor volume; all tumors progressed rapidly. While tumor stasis was achieved with 0.3 mg/kg RG7116 in mice with the smallest baseline tumors in both studies, only partial tumor growth control was achieved at this dose in the mouse with the largest baseline tumor volume (217 mm^3^) in study FaDu_001 (Fig. 4c). Interestingly, in FaDu_001, the mouse with the smallest baseline tumor showed an accumulation of RG7116 serum levels from the first administration (*C*_min_ = 1.94 µg/mL) to third administration (*C*_min_ = 2.67 µg/mL). However, the mouse with the largest baseline tumor volume did not show RG7116 accumulation levels during the dosing period (*C*_min_ = 1.44 µg/mL after first administration and *C*_min_ = 1.50 µg/mL following third administration). In study FaDu_008, the mouse with the largest baseline tumor volume (312 mm^3^) showed a tumor growth profile similar to the mean tumor growth profile from the mice treated with vehicle control (Fig. 4d), whereas the mouse with the smallest baseline tumor achieved tumor stasis during the length of the study. Similarly to mice in study FaDu_001, the mouse with the smallest baseline tumor showed higher *C*_min_ (1.62 µg/mL) 21 days after the start of dosing, whereas the mouse with the largest baseline tumor showed lower *C*_min_ (0.32 µg/mL).

### Expression of HER3 and pHER3 is modulated in skin and oral mucosa

**Fig. 5:**
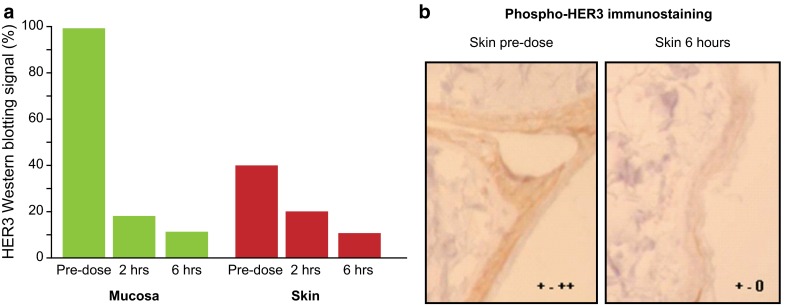
Down-modulation of HER3 and inhibition of HER3 phosphorylation in surrogate tissues. **a** Compared to baseline, levels of HER3 were markedly diminished in oral mucosa and skin biopsies at 2 and 6 h post-treatment in a cynomolgus monkey treated with 20 mg/kg RG7116. **b** IHC shows expression of pHER3 in skin tissue and was also reduced following treatment. (IHC was scored from 0 to +++; + to ++ indicates positive staining and 0 to + indicates borderline/negative staining)

As a surrogate approach, the ability to assess the effects of a therapeutic compound in tissue biopsies that are more easily accessible than the tumor would greatly facilitate future drug development and the assessment of pharmacodynamic effects in patients. Therefore, the potential for using oral mucosa and skin as surrogate tissues for pharmacodynamic marker analysis was investigated in a single cynomolgus monkey. This study used a higher dose (20 mg/kg) of RG7116 than was used in the murine models to ensure that both HER3 inhibition and 100 % target saturation were achieved. Following a single treatment with 20 mg/kg RG7116, the level of HER3 detected by Western blotting was dramatically reduced in mucosa and skin biopsies taken 2 and 6 h post-dose, compared to pretreatment levels (Fig. 5a). Expression of pHER3 in skin as measured by IHC was also markedly reduced (Fig. 5b) and was barely detectable at 6 h.

## Discussion

RG7116 is a novel anti-HER3 antibody that can exert a therapeutic effect through both the inhibition of HER3 signaling and by recruiting immune effector cells for ADCC [22]. In order to better understand the therapeutic potential of RG7116, we further assessed the anti-tumoral efficacy mediated by HER3-signaling inhibition in a FaDu cell line-based xenograft of human hypopharyngeal cancer and characterized the PK and PD changes occurring in response to treatment with RG7116 at different doses and dosing schedules.

RG7116 showed nonlinear PK in mice with rapid serum clearance at low doses (0.3–1 mg/kg) and slower clearance at higher doses (3 mg/kg). Systemic exposure (AUC) showed a greater than dose-proportional increase accompanied by a decline in total clearance with increasing dose, indicating that elimination of RG7116 is target-mediated [27]. Maximal efficacy was seen at doses ≥3 mg/kg, consistent with the slower clearance observed at that dose. In addition, the lack of RG7116 *C*_min_ accumulation following chronic administration of 0.3 mg/kg indicates a predominant involvement of target-mediated clearance resulting in suboptimal efficacy. Accumulation of RG7116 in serum was observed with doses from 1 to 10 mg/kg in study FaDu_001 and following 3 mg/kg administration in study FaDu_008. This distinct exposure pattern was consistent with the improved efficacy and greater decrease in the pHER3/HER3 ratio seen with higher doses, and is likely due to the target saturation. In addition, the PK profile of RG7116 was correlated with the pHER3/HER3 ratio profile following single-dose administration, with the faster elimination shown in the lower dose group (0.3 mg/kg) being consistent with an earlier return of pHER3/HER3 to basal levels.

This cell line-based tumor model is characterized by a rapid and progressive tumor growth in vivo indicated by a short tumor doubling time of 2–3 days. Therefore, randomization is done between Days 14 and 18 with individual variability, and control-treated animals have to be excluded early on, as is often seen with other models. Despite these model imitations, the anti-tumor efficacy induced by RG7116 in the FaDu xenograft model was strong and dose-dependent in both multiple treatment studies and consistent with previous data [22]. Efficacious doses of RG7116 also inhibited HER3 phosphorylation and down-modulated membrane HER3 expression in explanted tumor tissue in a similar manner as reported previously. Furthermore, dose-related downstream inhibition of pAKT was also observed. In all studies, the 3 mg/kg dose appeared to be optimal for the FaDu subcutaneous xenograft model system investigated. At this dose level, tumor stasis was achieved with weekly RG7116 in both multiple dosing studies, and target modulation was observed. No difference in efficacy was seen between the 3 and 10 mg/kg doses, suggesting that higher doses do not appear to offer further benefit. A lesser degree of tumor growth inhibition was seen with a suboptimal dose (0.3 mg/kg); however, rechallenging mice treated at this level with a higher dose (5 mg/kg) appeared to restore anti-tumor efficacy. Efficacy with 3 mg/kg given weekly was similar to that of 3 mg/kg given on a 3-week cycle. However, pHER3/HER3 was only ~50 % inhibited by the end of the q3w dosing interval (day 21). Further work is required to understand the extent of pHER3/HER3 inhibition required to achieve efficacy.

Despite initial tumor stasis, tumor regrowth was observed in mice treated once-weekly with 3 mg/kg RG7116 after approximately 46 days. This escape from RG7116-induced growth inhibition was associated with upregulation of both HER1 and HER2. Therefore, two reasons for failure to control tumor growth with RG7116 were observed: suboptimal dosing and activation of alternative signaling pathways involving HER1/2. Combined inhibition of HER3 and other members of the HER family is an attractive option for enhancing the efficacy of RG7116. The availability of potent mAb inhibitors of individual members of the HER family will allow for a personalized medicine approach in the clinic, with appropriate antibodies selected based on the HER profile of the patient’s tumor. This could also imply that a detailed molecular characterization of the tumor needs to be addressed at the time of progression to overcome bypass mechanisms. We previously demonstrated that combining RG7116 (anti-HER3 antibody) with RG7160 (anti-HER1 antibody) or pertuzumab (anti-HER2) achieved complete and long-lasting tumor growth inhibition in murine subcutaneous xenograft models in which tumor growth was driven by HER1 or HER2, respectively [22].

Inhibition of HER3 signaling alone was insufficient to control tumor growth in the immune-deficient mouse model used here. Additional efficacy is expected in humans through RG7116-mediated ADCC. RG7116 binds with high affinity to the FcRγIIIa receptor found on ADCC-competent cells such as macrophages and natural killer (NK) cells (absent in the SCID-beige mice used in these studies) and significantly enhances in vitro ADCC compared to non-glycoengineered RG7116 [22]. RG7116-mediated cell killing may also help suppress the development of resistance to HER3 signaling inhibition. Assessing the ADCC potential of novel humanized mAbs in animal models is challenging, as this requires the presence of immune effector cells (i.e., immunocompetent mice) and also that the human mAb interacts with the receptors on these cells. A SCID–human FcγRIIIa transgenic mouse model (which bears murine FcγRIV-positive macrophages and human FcγRIIIa-positive transgenic murine NK cells) was recently used to demonstrate the ADCC activity of imgatuzumab, which is glycoengineered in the same way as RG7116 [28]. A NOD/SCID/gamma c(null) mouse model, which is receptive to the administration of human NK cells, has also been developed to specifically investigate NK cell-mediated ADCC [29]. Further evaluation of RG7116 in models such as these is clearly warranted to investigate the contribution of RG7116-mediated ADCC to its efficacy.

RG7116-induced effects on HER3 and pHER3 levels were also seen in the skin and oral mucosa of a cynomolgus monkey, suggesting that these may be useful surrogate tissues for PD evaluation of RG7116 in early development clinical trials. Initial data from a first-in-human trial of RG7116 have shown that downregulation of membrane HER3 is also seen in skin samples of patients with HER3-positive epithelial tumors treated with RG7116 (at a dose of 100 mg and above), and this was associated with downregulation of HER3 in on-treatment tumor samples (at 200 mg and above) [30]. Skin biopsies have been used as a surrogate tissue for monitoring the effects of anti-HER1 therapies [31–34]. The value of this approach has been shown for erlotinib, where suppression of HER1 phosphorylation in the skin of patients with head and neck cancer was correlated with increased survival [35].

While these studies were not designed to address differences among baseline tumor volumes, response, and dose/exposure, we analyzed different mice representing animals with the smallest and highest tumor volume at baseline from each of the multiple dosing studies in an attempt to understand the variability in anti-tumor activity observed primarily in mice treated with the lowest dose (0.3 mg/kg). This dose was efficacious in animals with smallest baseline tumor volumes, but not in animals with larger tumors. The efficacy observed was consistent with the *C*_min_ accumulation observed in the mouse with the smallest tumor volume compared to no RG7116 accumulation observed in the mouse with the largest tumor volume. Such a finding might indicate that baseline tumor characteristics affect the efficacy of RG7116 at a given dose. Similar findings have been reported with the anti-CD20 mAb rituximab, where baseline tumor burden was correlated with survival in mice treated at the same dose [36]. Notably, size-matched FaDu tumor-bearing mice belonging to the vehicle group displayed progressive growth independent of their baseline size. However, further work will be required to properly explore the association between tumor volume, response, and dose for the RG7116 molecule.

In summary, RG7116 demonstrated strong dose-dependent tumor growth inhibition in a rapidly growing FaDu xenograft model of human cancer. Promising preclinical efficacy was demonstrated, and target modulation of pHER3 and HER3 was observed following weekly administration and with administration once every 3 weeks. These studies highlight the value of investigating pharmacokinetic behavior and measuring total HER3 or pHER3/HER3 ratio in tumor, and the value of skin biopsies as potential surrogate markers for efficacy and guiding optimal dosing in the clinic.
